# A facile *in vitro* model to study rapid mineralization in bone tissues

**DOI:** 10.1186/1475-925X-13-136

**Published:** 2014-09-16

**Authors:** Anthony J Deegan, Halil M Aydin, Bin Hu, Sandeep Konduru, Jan Herman Kuiper, Ying Yang

**Affiliations:** Institute for Science and Technology in Medicine, School of Medicine, Keele University, Stoke-on-Trent, ST4 7QB UK; Environmental Engineering Department & Bioengineering Division and Center for Bioengineering, Hacettepe University, Ankara, 06800 Turkey; Orthopedics Department, University Hospital of Staffordshire, Stoke-on-Trent, ST4 6QG UK

**Keywords:** Osteoblasts, Rapid bone formation, Matrix mineralization, Cell aggregates, Aggregate size

## Abstract

**Background:**

Mineralization in bone tissue involves stepwise cell-cell and cell-ECM interaction. Regulation of osteoblast culture microenvironments can tailor osteoblast proliferation and mineralization rate, and the quality and/or quantity of the final calcified tissue. An in vitro model to investigate the influencing factors is highly required.

**Methods:**

We developed a facile *in vitro* model in which an osteoblast cell line and aggregate culture (through the modification of culture well surfaces) were used to mimic intramembranous bone mineralization. The effect of culture environments including culture duration (up to 72 hours for rapid mineralization study) and aggregates size (monolayer culture as control) on mineralization rate and mineral quantity/quality were examined by osteogenic gene expression (PCR) and mineral markers (histological staining, SEM-EDX and micro-CT).

**Results:**

Two size aggregates (on average, large aggregates were 745 μm and small 79 μm) were obtained by the facile technique with high yield. Cells in aggregate culture generated visible and quantifiable mineralized matrix within 24 hours, whereas cells in monolayer failed to do so by 72 hours. The gene expression of important ECM molecules for bone formation including collagen type I, alkaline phosphatase, osteopontin and osteocalcin, varied temporally, differed between monolayer and aggregate cultures, and depended on aggregate size. Monolayer specimens stayed in a proliferation phase for the first 24 hours, and remained in matrix synthesis up to 72 hours; whereas the small aggregates were in the maturation phase for the first 24 and 48 hour cultures and then jumped to a mineralization phase at 72 hours. Large aggregates were in a mineralization phase at all these three time points and produced 36% larger bone nodules with a higher calcium content than those in the small aggregates after just 72 hours in culture.

**Conclusions:**

This study confirms that aggregate culture is sufficient to induce rapid mineralization and that aggregate size determines the mineralization rate. Mineral content depended on aggregate size and culture duration. Thus, our culture system may provide a good model to study regulation factors at different development phases of the osteoblastic lineage.

**Electronic supplementary material:**

The online version of this article (doi:10.1186/1475-925X-13-136) contains supplementary material, which is available to authorized users.

## Background

Rapid and effective repair of large bone defects and non-union fractures is still a challenging aspect in orthopaedic surgery. Without intervention at the defect sites, automatic bone formation in such areas frequently does not occur [1]. Gold standard interventions include the addition of bone grafts, the administration of growth factors or applying low-intensity pulsed ultrasound [1–3]. However, even these gold standard interventions are not without problems and bone tissue engineering techniques have thus attracted great attention. Animal models have demonstrated that tissue engineered bone could bridge bone defects or generate new bone around the defects [4, 5]. However, the time required for bone tissue regeneration within such models *in vitro* is relatively long. Thus, a need exists to develop a more effective and reliable model to study rapid generation of high quality autologous or allograft bone and define the influencing factors.

Bone has a unique capacity for regeneration with the process of bone repair following the same pathway as that during normal bone development [6, 7]. Direct or intramembranous bone development within the body is characterized by a complex cascade of reactions which orchestrate the initiation and ceasing of proliferation, the onset of differentiation and the beginning of mineralization [8, 9]. A critical challenge for *in vitro* bone formation, e.g. through a tissue engineering approach, is to create a cellular and matrix niche for bone cells or stem cells to follow this same pathway [10]. The ultimate goal for bone tissue engineering is to generate high quality and high quantity minerals *in vitro* within a short *in vitro* culture period, which can be implanted into the defect sites of hosts to initiate bone repair. In the past decades, the predominant procedures for bone tissue engineering have adopted a 2D (monolayer) culture approach [11]. Even when using 3D scaffolds, most cells within the porous scaffolds essentially grow in a monolayer manner along the internal scaffold surfaces for a prolonged period. Given that monolayer cultures of osteoblasts generally do not produce well-mineralized osteoid, or at best, require a prolonged culture time to generate bone nodules, such culture methods may require refinement.

Recent studies suggest that the use of a micromass approach in tissue engineering, which uses cellular aggregates instead of monolayer cultures, may offer a more productive solution for bone tissue engineering [12, 13]. When adopting this technique, cell aggregates have been formed by cultivation in shaker flasks, hanging drop cultures, 3D rotary wall vessel and on agarose coated multiple well plates [14–18]. 3D rotary cell culture is the better version of shaker flasks in bone tissue formation. These new *in vitro* culture techniques follow the hypothesis that cellular condensation and aggregate formation can greatly enhance bone tissue development by mimicking the intramembranous ossification pathway. In particular, these bone cell aggregates can and will become ossification centres for nodule development and can greatly reduce the time required for mineralization. During intramembranous bone formation, cell condensation or aggregation is an essential step for mineralization [19]. The cell-cell contact in aggregation induces several cellular events, in particular, proliferation arrest, terminal differentiation and the formation of an osteoid for the deposition of minerals [20]. It is well established that cell-cell contact and condensation and the presence of the correct protein-rich extracellular matrix are essential niches for bone formation [8, 21].

In this study we aim to further the aggregate culture principle by using substrate chemistry modifications to induce bone aggregate formation in large quantities and with variable sizes. We intend to develop a facile model enabling us to identify the variables that control the mineralization process and the quality of minerals formed in *in vitro* 3D aggregates. This will be done by examining gene expression profiles and mineral production rates whilst also evaluating mineral properties. We postulate that the outcome of the quantitative examination comparing culture environments and time may help to establish distinct models which are in different bone formation stages. Such models will allow for the investigation of parameters that dictate the quality and quantity of mineralization, important for generating implantable bone for clinical applications.

## Materials and methods

### Aggregate formation

#### Cell source

A late osteoblast murine cell line, MLO-A5 (passage number 26, kindly donated by Professor Lynda F Bonewald) [22] was used in this study. The cells were cultured in α-MEM (Invitrogen, UK) supplemented with 5% standard fetal bovine serum (sFBS) (Fisher, UK), 5% bovine calf serum (BCS) (Fisher, UK) and 1% antibiotic-antimycotic solution (A + A) (Sigma, UK) at 37°C and 5% CO_2_. The cells were enzymatically cleaved with trypsin (Lonza, Belgium) and passaged once they had become 80% confluent.

#### Modification of cell culture substrates

To generate different sizes of bone cell aggregates, two different cell culture substrates were used. The first was a commercially available 24-well polystyrene cell suspension plate (Sarstedt, UK), a hydrophobic surface denoted as non-coated. The second substrate was the same type of plate, modified with 20 mg/ml Pluronic F127 solution (BASF, USA), denoted as coated. To make coated plates, 500 μl of the Pluronic solution was added to each well of the 24-well polystyrene cell suspension plate and incubated at room temperature for 24 hours. The remaining solution was removed and the wells were dried in a biological safety cabinet for later usage. Standard 24 well cell-adhesive plates (Sarstedt, UK) were used to culture cells in monolayers.

#### Formation of bone cell aggregates

The bone aggregates were formed on non-coated and coated plates by seeding MLO-A5 cells into 24-well plates at a density of 3×10^5^ cells per well. The aggregates were cultured statically for 24, 48 and 72 hours. Monolayer cell culture on 24-well cell culture plates has been used as the control. To avoid the cells becoming confluent within the same culture duration (24, 48, 72 hours), the initial seeding densities for the monolayer cultures were set lower than 3×10^5^. They were 1.2×10^5^ for 24 hour samples, 1.0×10^5^ for 48 hour samples, and 0.8×10^5^ for 72 hour specimens. An osteogenic medium consisting of α-MEM supplemented with 5% sFBS, 5% BCS, 2 mM L-glutamine (Lonza, Belgium), 10 μg/ml ascorbic acid (Sigma, UK), 10 nM dexamethasone (Sigma, UK), 10 mM glycerophosphate (Sigma, UK), and 1% A + A was used without medium change during the entire culture period at 37°C and 5% CO_2_.

### Characterization of aggregates and minerals

#### Optical microscopic imaging

The aggregate formation was monitored by light microscopy (Olympus, UK) at indicated culture time points. The average aggregate size was measured based on images taken with at least three replicates from three separate experiments. After 48 hour initiation of cell aggregate formation, some of the formed aggregates were transferred into normal 6-well cell adhesive cell culture plates (Corning, USA) for an outgrowth study cultured in the osteogenic medium as stated above. The aggregates-outgrowth profile on the adhesive cell culture plate was recorded for 20 hours while under culture by Cell IQ equipment (Chip-Man Technologies, Finland).

#### Alkaline phosphatase staining

Alkaline phosphatase (ALP) staining was conducted using an ALP kit (Millipore, UK). The monolayer samples were fixed *in situ* using 4% paraformaldehyde for no more than 2 minutes and stained following the kit instructions. The aggregate samples were transferred to micro-centrifuge tubes and collected through centrifuging. The fixation and staining procedures were the same as those mentioned for monolayer specimens but in micro-centrifuge tubes as opposed to wells. The stained aggregates were transferred to slides for imaging.

#### Real-time PCR

For the monolayer samples, Tri Reagent lysis buffer was added to each well and pipetted. The lysed solutions were collected and stored at -80°C until analysis. The aggregated samples were transferred from their wells to micro-centrifuge tubes prior to lysing. Tri Reagent lysis buffer was then added to each of the micro-centrifuge tubes and the aggregate pellets were thoroughly pipetted and mixed to obtain the complete lysis before being stored in a -80°C freezer for further analysis. RNA extraction was carried out using the Tri Reagent protocol and was quantified using spectrophotometry (Nanodrop 2000), the results of which were then used to establish the required amounts of RNA for the next stage of the analysis. Reverse transcription was carried out using an RT kit (Quantitect RT, Quiagen) and cDNA synthesis followed manufacturers protocols. PCR analysis was carried out using qPCR (Mx3000P, Agilent Technologies, UK) with MxPro software. Gene 18S was used as the endogenous control for normalization of expression levels on the genes of interest using the delta delta CT (ΔΔ CT) method. Each ΔΔ CT value was used in the formula 2^-ΔΔCT^ to give a comparative fold change of gene expression level relative to that same gene in the monolayer sample at 24 hour culture. Four genes were analyzed: collagen type I (COL1), alkaline phosphatase (ALP), osteopontin (OPN), and osteocalcin (OCN). Each culture condition had three parallel specimens.

#### MicroCT/histological analysis

After the given culture duration, the cellular specimens were fixed for microCT and histological analysis. The monolayer samples were fixed *in situ* using 100% ethanol. Prior to fixing, the aggregate samples were transferred to micro-centrifuge tubes and fixed with 100% ethanol. Both monolayer and aggregate samples were stored at 4°C for further analysis.

Aggregate specimens were encapsulated in 0.2 ml of 3 mg/ml collagen hydrogel before conducting microCT, to facilitate handling of the small sized specimens. An identical blank hydrogel was used as a control to define the threshold for bone tissue analysis. The microCT scanning was carried out at a resolution of 8 μm using the provided software and protocols by the manufacturer (μCT40, Scanco Medical AG, Switzerland).

Once the microCT scans were completed, the same samples were used for histological analysis. A standard wax embedding procedure was adopted and 5 μm thick slices were used. An Alizarin red staining protocol was used to evaluate the calcium content. Alizarin red solution (1% w/v, pH 4.1) was prepared freshly in deionized water and filtered through a 0.22 μm filter. The samples were covered in solution and left at room temperature for 40 minutes then were thoroughly washed with deionized H_2_O. For von Kossa staining, the samples were covered with 5% silver nitrate and incubated for 30 minutes at 37°C. Then the samples were thoroughly rinsed with deionized H_2_O and incubated with 5% sodium carbonate (in 25% formaldehyde) for 5 minutes at room temperature. The samples were then ready for imaging.

#### SEM-EDX analysis

The mineral distribution within the aggregates was analyzed using SEM in combination with energy dispersive X-ray analysis (SEM-EDX) (TM-3000, Hitachi, Japan). The fixed aggregates were embedded in cryoprotectant OCT compound (Tissue-Tek®; Sakura Finetek, UK) and cut into thin sections of 10 μm using a cryostat-microtome (Thermo Shandon, UK). The elemental distribution of Ca and P within the aggregates was mapped and analyzed.

#### Statistics

Three specimens per group were tested. Recorded data was averaged and represented as a mean value ± SEM. Groups were compared using independent T-tests or one-way analysis of variance (ANOVA). A p-value below p = 0.05 was denoted to indicate statistical significance. In graphs, statistical significance is indicated at three levels: *p ≤ 0.05, **p ≤ 0.01, and ***p ≤ 0.001.

## Results

### Aggregate morphology and size

**Figure 1:**
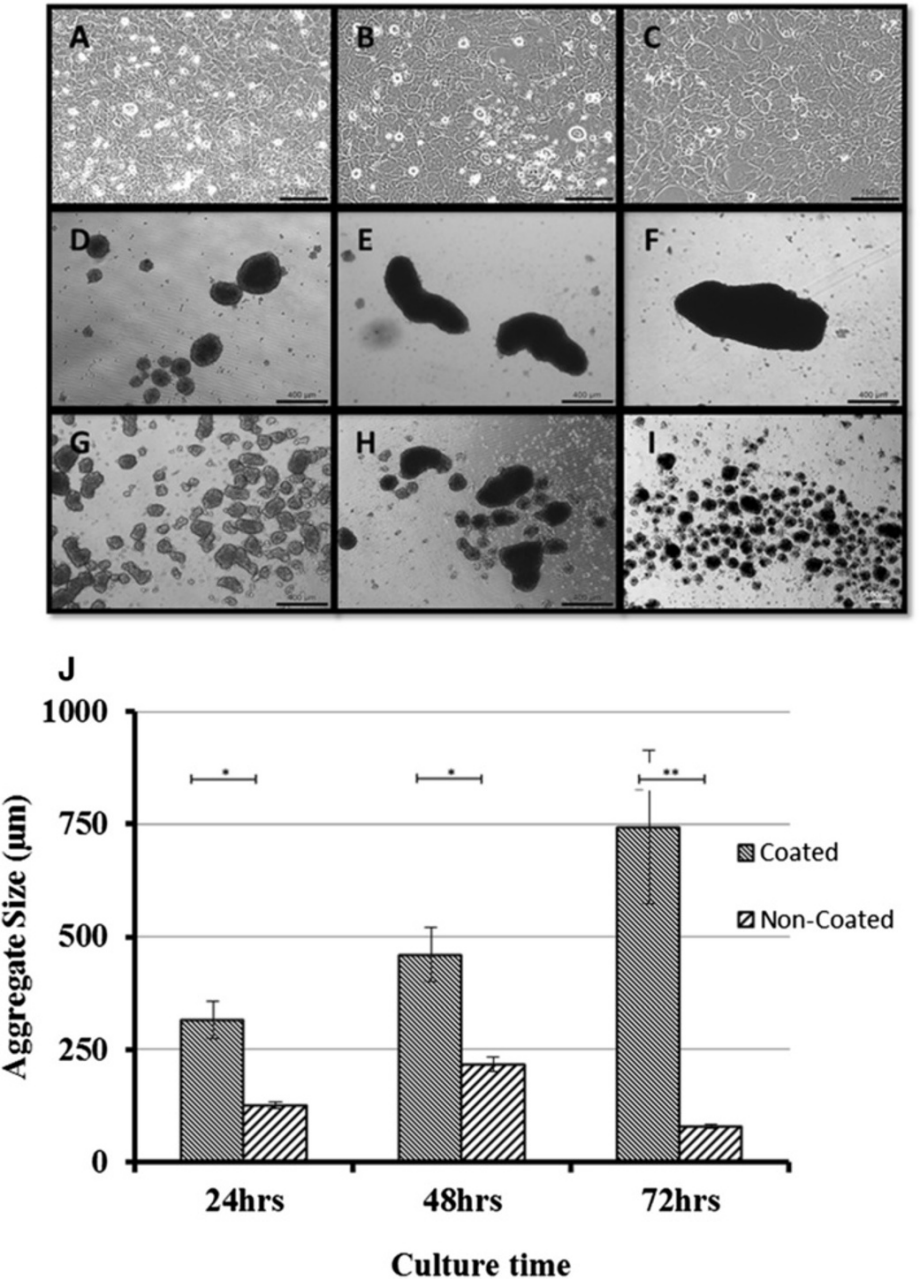
**Optical images taken for the three types of cultures (A-C: monolayer; D-F: coated substrate; G-I: non-coated substrate) over three culture time points (A, D, G: 24 hours; B, E, H: 48 hours; C, F, I: 72 hours). J** is the graph showing the average size of the aggregates produced on both coated and non-coated substrates over three time points; 24, 48, and 72 hours. Measurements were taken in triplicate (minimum). Error bars represent SEM. Scale bar in figures A-C is 150 μm; D-G 400 μm.

Aggregation on both coated and non-coated culture substrates was observed to occur within just hours of suspension culturing. The size and shape of the aggregates formed showed a large variation, with considerable differences already evident after 24 hours culturing. The aggregates formed within the first 24 hours in non-coated suspension culture did not clump together and remained separate and uniformly ‘spheroidal’ in shape, unlike those in coated suspension culture (Figure 1D, G). Those formed in coated suspension culture were almost three times larger than those in non-coated suspension culture (t-test, p = 0.005). By 48 hours, the average size of the non-coated suspension aggregates had ceased to increase (Figure 1H) and by 72 hours they had started to condense and shrink in size (Figure 1I). In contrast, continuous aggregate growth was noted in the coated suspension cultures up to the 72 hour time point (Figure 1E, F). The increased aggregate size of the coated suspension cultures appeared to be caused by smaller aggregates joining together and forming large, ellipse shaped aggregates (Figure 1F). At 72 hours, the average size of aggregates was 79 μm for those formed on non-coated substrates and 745 μm for those formed on coated substrates (Figure 1J). At 72 hours, the number of aggregates obtained varied between approximately 20 and 30 per well for the non-coated plates, and between 2 and 3 per well for the coated plates.

After transferring to adhesive plates aggregates cultured for 48 hours on suspension plates, some cells migrated out or proliferated from the periphery of aggregates and formed a sunflower-shaped morphology. Interestingly, small aggregates turned into planar cell plaque and lost the dense central part during the outgrowth stage, while the larger aggregates maintained the dense central part unchanged. (Additional file 1, aggregate outgrowth video). A crystal-like solid had formed at 10 day culture (data not shown).

### Gene expression

**Figure 2:**
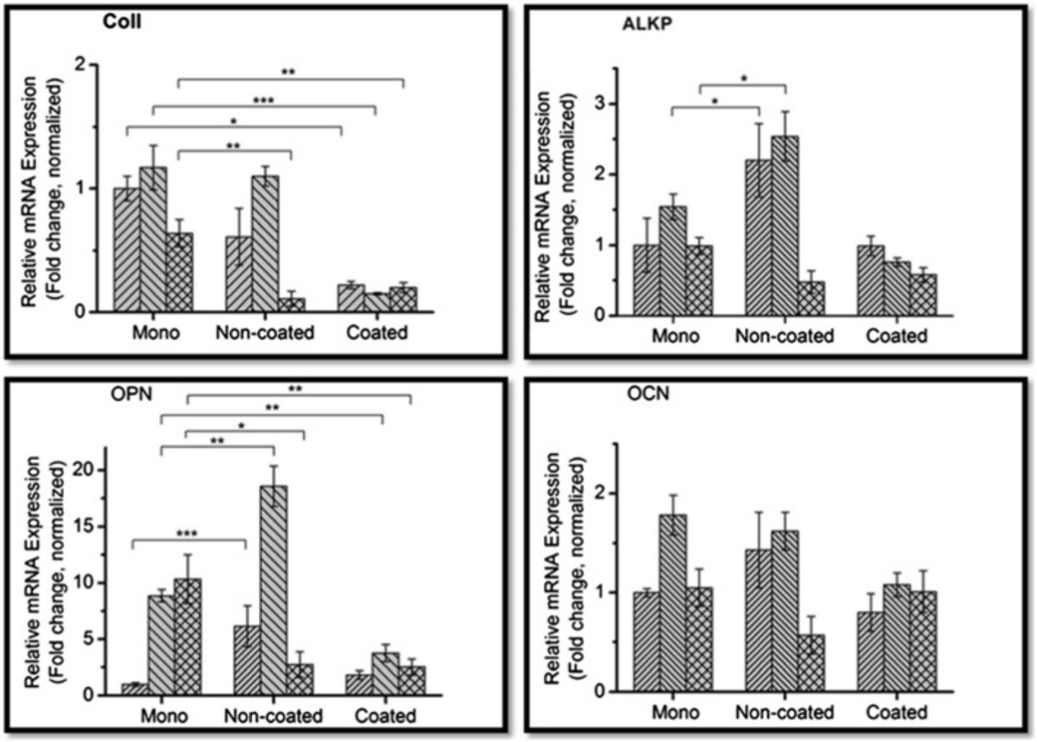
**The expression levels of four different genes associated with osteogenesis; collagen type I (COL1), osteopontin (OPN), alkaline phosphatase (ALP), osteocalcin (OCN) as the function of the culture conditions and time duration.**

The four genes; COL1, ALP, OPN and OCN were expressed under all three cell culture conditions, but the expression pattern varied by culture duration and culture substrate. Except for COL1, non-coated aggregates had higher gene expression levels than the other two groups (Figure 2).

Collagen type I (COLl): At 24 and 48 hours, mean collagen expression levels in the monolayer and non-coated suspension culture specimens were higher than in the coated suspension culture specimens. At 24 hours, the monolayer culture had a 4.5 fold increase (95% CI 3.04 – 7.19) over the coated suspension culture specimens, whilst the non-coated suspension culture specimens had a 2.7 fold increase (95% CI 0.21 – 6.02) over the coated suspension culture specimens. At 48 hours, the monolayer culture had a 7.8 fold increase (95% CI 4.76 – 11.26) in COL1 expression over the coated suspension culture specimens, whilst the non-coated suspension culture specimens had a 7.3 fold increase (95% CI 5.72 – 9.33) over their coated suspension counterparts. At 72 hours, the mean expression level in the non-coated suspension culture specimens had dropped to that of the coated suspension culture specimens, whereas mean expression levels remained relatively high in the monolayer culture specimens with a 5.8 fold increase (95% CI N/A) over the non-coated suspension culture specimens and a 3.2 fold increase (95% CI 1.63 – 6.78) over the coated suspension culture specimens.

Osteopontin (OPN): The three culture conditions exhibited dramatically different expression patterns. The coated suspension culture maintained a relatively constant expression for all three time points, whilst the monolayer and non-coated suspension cultures experienced changes in expression throughout the culture period. At 24 and 48 hours, the non-coated suspension culture specimens expressed a 6 fold increase (95% CI 1.63 – 13.21) and 2 fold increase (95% CI 1.54 – 2.73), respectively, over the monolayer specimens, which in their turn expressed a 2.35 fold increase (95% CI 1.52 – 4.71) and a 4 fold increase (95% CI 2.68 – 6.5) over the coated suspension culture specimens at 48 at 72 hours, respectively (Figure 2). The peak expression along the culture time points was different in the three types of specimens. The peak expression in both aggregate cultures was at 48 hours, whereas that in the monolayer culture was at 72 hours.

Alkaline phosphatase (ALP): At 24 and 48 hours, aggregates formed in non-coated suspension culture expressed the highest levels compared to the other two groups with a 2.2 fold increase (95% CI 0.75 – 31.74) at 24 hours and a 1.6 fold increase (95% CI 1.02 – 2.57) at 48 hours. While the monolayer and non-coated suspension culture specimens had their peak ALP expressions at 48 hours, the coated suspension culture specimens had their peak expression at 24 hours and continuously decreased thereafter.

Osteocalcin (OCN): The mean expression levels of OCN were similar in the three groups, with the highest mean expression in all three groups seen at 48 hours. At 72 hours the non-coated suspension culture specimens exhibited the lowest mean expression levels amongst the three groups with a 0.5 fold increase (95% CI 0.09 – 1.25) compared to both the monolayer and the coated suspension culture specimens. The coated suspension culture specimens showed quite similar levels throughout the three culture points.

### Histochemical staining

**Figure 3:**
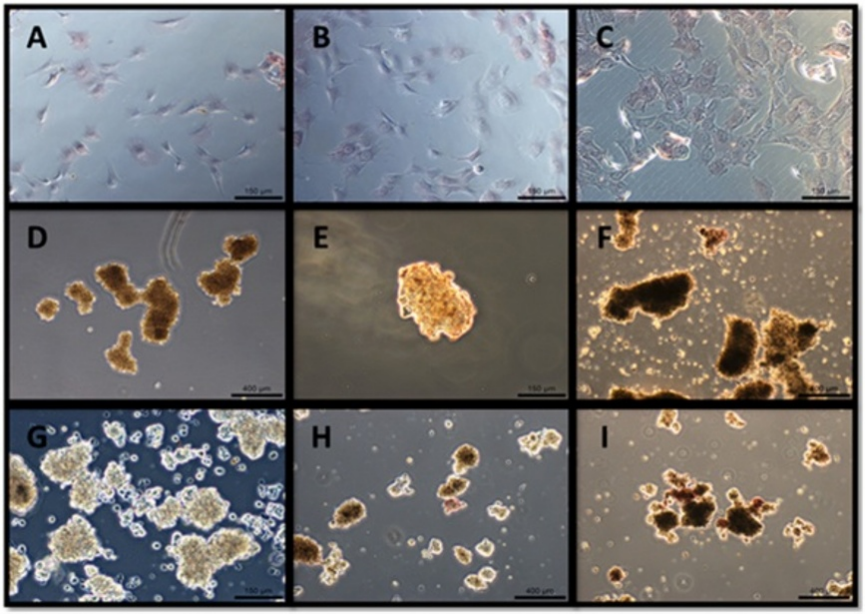
**ALP activity of the cells in the three types of cultures (A-C: monolayer; D-F: coated substrate; G-I: non-coated substrate) over three culture time points (A, D, G: 24 hours; B, E, H: 48 hours; C, F, I: 72 hours).** The scale bars of figures A-C, E, G are 150 μm; the rest figures 400 μm.

**Figure 4:**
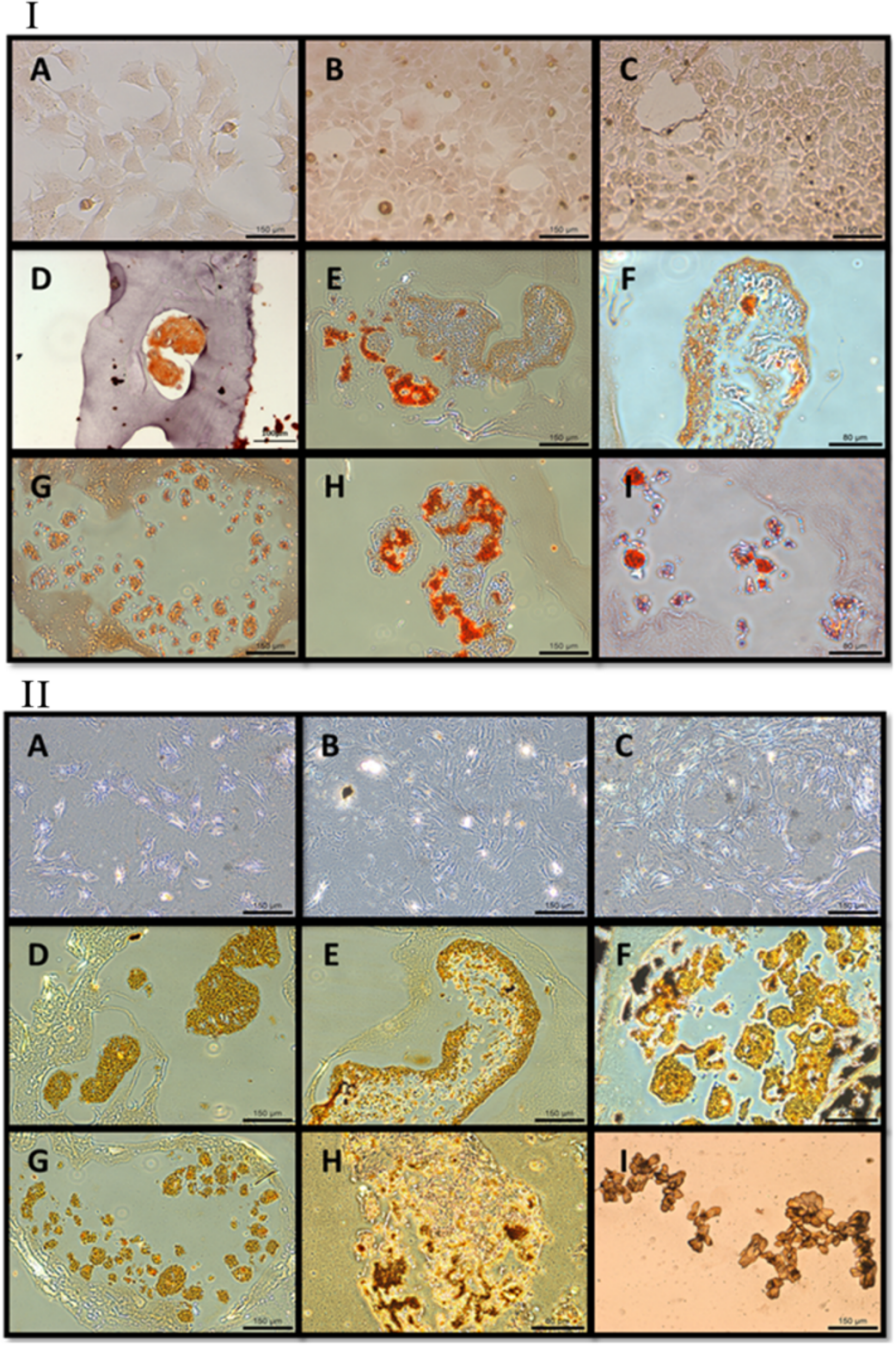
**Alizarin Red staining (I) and von Kossa staining (II) of the cells in the three types of cultures (A-C: monolayer; D-F: coated substrate; G-I: non-coated substrate) over three culture time points (A, D, G: 24 hours; B, E, H: 48 hours; C, F, I: 72 hours).**

At all three time points, the monolayer culture specimens showed marginal ALP staining. At 24 hours, both types of aggregates showed faint staining, and at 48 and 72 hours, they stained positively (Figure 3). Monolayer culture specimens did not show positive Alizarin Red or von Kossa staining at all, whereas all aggregates, whether cultured on coated or non-coated substrates, stained positively with both Alizarin Red and von Kossa (Figure 4). The strongest von Kossa staining was on the 72 hour coated suspension culture specimens. The encapsulating collagen was not stained by the Alizarin Red or von Kossa dyes, indicating that physical entrapment of the dye did not result in false positive staining.

### MicroCT

**Figure 5:**
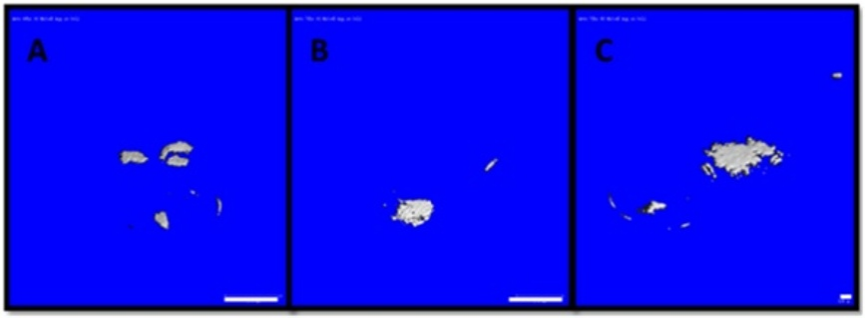
**MicroCT images of aggregates at a threshold of 130. A)** from the coated substrate at 48 hours culture; **B)** from the non-coated substrate at 72 hours culture; **C)** from the coated substrate at 72 hours culture. Scale bar in images A and B = 1 mm, image C = 100 μm.

The mineral content in both types of aggregates was evaluated by using a predetermined threshold on microCT scan images. A threshold of 100 was used as the benchmark, set by the collagen hydrogel in which the aggregate samples were encapsulated. Figure 5 shows microCT scan images of aggregates at a threshold of 130. At 24 hours culture, none of the aggregates appeared denser than the collagen hydrogel. At 48 hours, the aggregates formed on coated suspension cultures appeared denser than the collagen hydrogel in which they were encapsulated. At 72 hours, both types of aggregates appeared denser than the collagen hydrogel. These aggregates continued to be visible up to a threshold of 160. It was impossible to evaluate monolayer culture specimens with microCT. However, the negative histological staining combined with the absence of nodule formation within the monolayer culture specimens demonstrated that there was no mineral content in these specimens.

### SEM and elemental analysis

**Figure 6:**
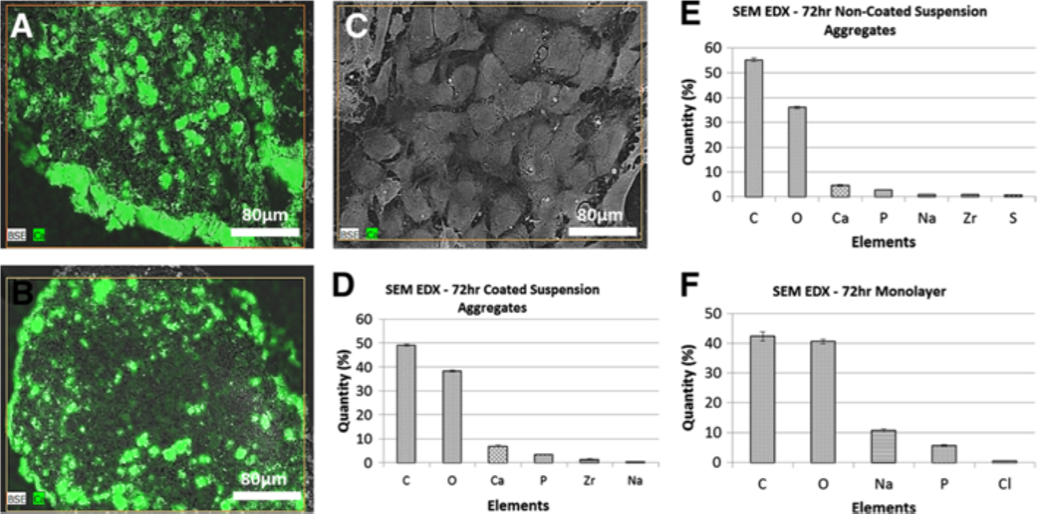
**SEM-EDX images and element analysis for the cells in the three types of cultures at 72 hour. A)**, **D)**: coated suspension culture; **B)**, **E)**: non-coated suspension culture; **C)**, **F)**: monolayer. **G** is the average size of the CaP nodules within aggregates (t-test, p = 0.0001).

SEM revealed large differences between the morphologies of aggregated and monolayer cultures. Both types of aggregates consisted of numerous nodules surrounded by thick peripheral layers, while in monolayer specimens individual cellular cytoskeletons were visible (Figure 6). The elemental analysis demonstrated that the three types of specimens had very similar carbon to oxygen ratios (1.02, 1.01, 1.12 for coated, non-coated and monolayer, respectively). Only the nodules contained both calcium and phosphate, with co-location distributions. The ratio of calcium and phosphate levels differed between aggregates cultured on coated and non-coated substrates. Aggregates from coated substrates had a higher calcium content, whereas aggregates on non-coated substrates had a higher phosphate content. Interestedly, the Ca:P weight ratio in the nodules from the big aggregates was 2.58, slightly higher than the theoretical Ca:P weight ratio of hydroxyapatite (2.15) [23], whilst the small aggregates had a lower ratio value, 1.74, than the theoretical one. The number of nodules within the aggregates did not differ much between the coated and non-coated suspension culture specimens, however the average size of nodules in coated specimens was 36% larger than those in non-coated specimens (Figure 6G).

## Discussion

In this study, we established a facile bone cell aggregation model by using a simple and convenient surface modification technique to promote the production of different sized bone aggregates. We confirmed the acceleration effect of mineral formation in aggregates compared to a monolayer culture. Consistent with reports from other groups [11, 24], our data demonstrates that culturing osteoblasts in aggregate cultures generated far more mineralized matrix in identical culture medium and duration than culturing them in monolayer culture, where they generated little or no mineralized matrix. The experimental results revealed that the gene expression of important ECM molecules for bone formation, including COL1, ALP, OPN and OCN, was temporally and spatially regulated, most likely influenced by cell-cell and cell-ECM interactions. In addition, the mineral content and composition within the bone aggregates depends on the culture substrate (coated vs. non-coated), evidenced by the elemental analysis and histochemical staining of the aggregates. We postulate that aggregate culture mimics the endogenous microenvironment thus supporting osteoblast survival and full osteogenic differentiation [25]. An important effect of the culture substrate was on aggregate size. Aggregation pushes osteoblasts from a proliferation phase to a matrix synthesis, or maturation and mineralization phase, whilst aggregate size determines how fast and how far these cells can be driven toward mineralization of the matrix.

Our study shows that altering the chemistry of culture well substrates is a convenient and efficient technique to promote cell aggregation. Multiple aggregates can be formed within just two hours, without the requirement of a complicated mould or hanging drop culture. We used two different substrates to control aggregate size, i.e. an uncoated polystyrene surface and a Pluronic F127 coated surface. Pluronic F127 is a polypropylene oxide-polyethylene oxide (PPO-PEO) tri-block copolymer with two hydrophilic ethylene oxide side chains and a hydrophobic propylene oxide chain in the middle [26], which endows Pluronic F127 with extremely flexible molecular chains and a high capacity to hydration. Coating substrates with F127 greatly suppresses protein adhesion [27, 28], preventing seeded cells from adhering to the substrate and inducing them to form aggregates which remain in suspension. The larger aggregates found in the F127 coated plates were ascribed to the higher expulsion force of F127 for proteins [27, 28]. These aggregates were spheroidal rather than spherical. Our data reveal that aggregate size, controlled by substrate chemistry, affected the mineralization rate. This finding is consistent with an earlier study on a spheroid culture system with different cell numbers in each aggregate [29]. In that study, higher cell numbers resulted in larger aggregates that produced higher calcium contents, a similar tendency to our results. Our microCT and SEM-EDX data seem to indicate that the size of the aggregates may be a critical parameter in controlling calcification rate and mass. By tailoring the substrate composition, we can generate different sizes of aggregates, enabling the study of different mineralization stages.

**Figure 7:**
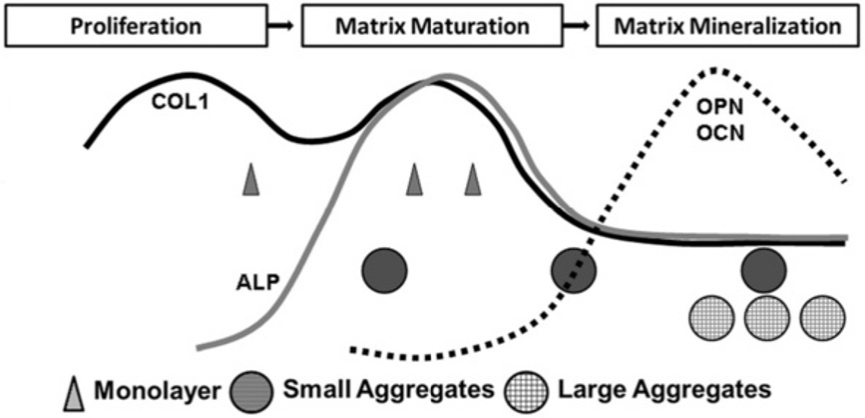
**Prediction of the osteoblast development stage as the function of the culture conditions and duration through depicting the temporal gene expression data (Figure** 2 **) into the profile of marker gene expression produced by Lian and Stein (modification of Figure** 4-3 **in** **[**[32]**])** **.** It is indicated that the monolayer specimens () stayed in a proliferation phase for the first 24 hours culture, and remained in matrix synthesis phase for the following 48 and 72 hours culture; whereas the small aggregates () were in the maturation phase for the first 24 and 48 hours culture and had jumped to a mineralization phase at 72 hours. Large aggregates () were in a mineralization phase at all three culture time points (24, 48 and 72 hours).

*In vivo* bone formation is a prolonged process characterized by a series of reactions. Osteoblasts play a pivotal role in all stages. Osteoblasts are derived from osteoprogenitor cells, frequently from pluripotent mesenchymal stem cells [30]. The major role of osteoblasts is to produce extracellular matrix proteins and to regulate the mineralization of the matrix. Gene expression patterns for these ECM proteins are similar between the *in vivo* developmental sequence and *in vitro* culture systems [31]. The rapid expression of ALP and collagen is followed by mineralization [31, 32]. Fibronectin and COL1 levels are high during proliferation and decline in the differentiation stage. However, it has been reported that OPN expression is slightly different *in vivo* and *in vitro*: *in vivo* levels continue to increase after animal birth while *in vitro* levels decrease when reaching the calcification stage. Nevertheless, osteoblast differentiation takes place in sequential steps. Lian and Stein [9, 32] have depicted the temporal expression of cell growth and osteoblast phenotype related genes during monolayer culture experiments and used them to define three distinct phases of osteoblast phenotype development eventually leading to bone formation, namely (1) proliferation and matrix synthesis; (2) matrix development, maturation and organization; and (3) matrix mineralization (Figure 7) [9, 20, 32, 33]. Interestingly, when we plot the gene expression data of our three types of cell cultures under different culture durations on the same figure, a distinct separation between the aggregates and monolayer culture methods was suggested (Figure 7). The monolayer culture specimens seemed to stay in the late proliferation/early matrix maturation phase for the first time points tested and for the last two time point remained in matrix maturation, not mineralization. However, the small aggregates seemed to be in the maturation phase for the first two time points and had moved to the mineralization phase by the third time point. In contrast, the large aggregates seemed to be in the mineralization phase for all three time points.

In this study, the monolayer culture exhibited the highest COL1 expression for all three time points. A high COL1 gene expression level suggests that the monolayer culture specimens were in a late proliferation/matrix maturation phase (see also Figure 7) [9, 32–37]. There are two peaks across the two phases [32], implying collagen production lasts a long period in the development pathway. The large aggregates (coated suspension culture) demonstrated a low expression of COL1 and ALP, suggesting that they had left the maturation phase within just 24 hours of culturing and started transferring to the mineralization phase (Figure 7). OPN expression in the monolayer culture continuously increased, further suggesting that they were in a late proliferation/maturation phase, whilst in both aggregate cultures OPN expression was down-regulated at the 72 hour time point, reinforcing the suggestion that these cultures had entered the mineralization stage (Figure 7). Osteocalcin has been classified as the latest of expression markers in mature osteoblasts [38]. Our OCN expression was similar in all culture specimens, suggesting all had at least reached a late osteoblast stage.

Other outcomes of this study, including protein and mineral productions, support the above phase mapping (proliferation, maturation, and mineralization) of the specimens based on gene expression. The SEM-EDX, microCT, and histological stains all indicate that coated suspension culture specimens produced larger aggregates which contained a higher quantity of minerals than non-coated suspension culture specimens, which were smaller aggregates. This was probably due to the coated suspension culture specimens entering the matrix mineralization phase earlier and staying there for a longer duration during the 72 hour culture period, compared to the non-coated suspension culture specimens. Monolayer culture specimens, on the other hand, had minimal mineralized extracellular matrix, suggesting that they did not enter the mineralization phase. Both the SEM-EDX and microCT data showed higher levels of mineralization within the aggregate cultures, with the SEM data in particular offering some interesting insights into the elemental make-up of the aggregates. Visually, it appeared that the larger aggregates from the coated suspension cultures had higher levels of mineralization. These aggregates had higher levels of calcium and lower levels of phosphate, compared to the smaller aggregates of the non-coated suspension cultures. The monolayer cultures stained negatively with Alizarin Red and von Kossa at all three culture points (Figure 4) indicating an absence of calcium and phosphate ions, and therefore mineralization. The aggregates from both coated and non-coated suspension cultures did stain positively with both Alizarin Red and von Kossa, indicating the presence of calcium phosphate. The co-localization of calcium and phosphorus was confirmed by SEM-EDX (Figure 6).

From our study and those reported by other groups, it becomes clear that monolayer culture of even late osteoblasts under conditions of non-confluency maintains them in that state and could not drive them into matrix mineralization for the duration of our experiments (3 days, see also Kato *et al*. [22]), whilst aggregate culture can easily and decisively drive this later proliferation/early maturation phase into the subsequent mineralization phase. A unique aspect of our method is to finely control the stage of the osteoblast population along the osteoblast developmental sequence. If osteoblasts are cultured into aggregates of a large size by using coated suspension culture wells, they have a high probability of quickly passing the late proliferation and maturation phase and entering the matrix mineralization phase. Within such a culture environment, a large portion of the cell population has intense cell-cell contact, inhibiting further proliferation and causing a jump to the subsequent phase. In the smaller aggregates produced using uncoated suspension culture wells, a large proportion of the cell population is lacking intense cell-cell contact, in particular the cells on the periphery of the aggregates. These cells therefore remain in the matrix maturation phase for a longer period, before eventually entering the matrix mineralization phase at 72 hours. Based on the data from this study, a large aggregate size can be defined as ranging from 500 to 1000 μm, whereas a small aggregate size ranges from 80 to 300 μm. Taking these analyses together, our results imply that when osteoblasts are kept in monolayer culture, even though they are late osteoblast phase cells, they will be kept in a proliferation/matrix maturation phase, expressing both proliferation and maturation markers. Further investigation is needed to define more accurately the aggregate size regions that determine which developmental phase is reached within a 72 hour culture period (indicated in Figure 7).

Cell viability is a critical issue in aggregate culture but it is difficult to accurately evaluate cell viability within the aggregate centers. Nevertheless, the fact that both types of aggregates showed an outgrowth capacity would suggest a high cell viability was maintained. The difference between the outgrowth profiles of small (non-coated culture) and large (coated culture) aggregates was maintaining their dense central part. The smaller aggregates were seen to outgrow to the point where they had lost the majority of their dense central regions, whereas the larger aggregates appeared to maintain their dense centers. It is postulated that the centers had a high concentration of minerals where intense cell-cell contact occurred in comparison to peripheral locations, which is of critical importance to forming tissues [39]. The cells within the larger aggregates are more likely to experience close cell-cell and cell-ECM interactions than those in the smaller aggregates simply because smaller aggregates have a relatively larger external surface. The cells on surface areas will have fewer cell-cell contacts. Once mineralized, the central part remained unchanged during culture.

Hypoxia is another factor postulated to encourage mineralization [40]. Hypoxia could be more prominent in the centres of large aggregates, thus explaining the relationship between aggregate size and mineralization. However, the role of hypoxia is controversial, with other studies demonstrating that hypoxic conditions down regulate osteogenic differentiation and subsequent mineralization in 3D micromass cultures of osteocytes [41, 42]. Oxygen and nutrient deficiency within aggregate cultures could lead to necrosis [43]. However, the histochemical staining and SEM analysis of the aggregates, both small and large, in our study showed no signs of necrosis or diminished osteogenic development within the aggregate centers. Differences in cell-cell interactions are thus more likely to explain the differences in level of mineralization between different sized aggregates found in this study.

A limitation of this study is its use of a pre-osteoblast/pre-osteocyte murine cell line MLO-A5, rather than primary osteoblasts. Primary bone cells or mesenchymal stem cells would be more relevant to test the relationship between cell aggregate size and mineralization rate and study its role *in vivo* or for clinical tissue engineering applications. However, MLO-A5 cell line has a few advantages than other osteoblast cell lines, mainly it produces high proteins of mineralization markers including alkaline phosphatase, osteocalcin, collagen, bone sialoprotein. The mineralization process of MLO-A5 cells has been characterized using a variety of high resolution microscopy techniques [44–46]. The mineralization mechanism in MLO-A5 has been reported similar to those observed during lamellar bone formation [44]. We therefore believe that our current results do have relevance to the *in vivo* process. Investigations are currently underway to ascertain whether these same findings can be obtained using primary cells and repeated *in vivo*.

## Conclusions

In conclusion, by modifying culture substrates, this study generated a convenient and effective model to delineate the key regulators of mineralization. This study clearly indicates that it is possible to shorten or extend either the proliferation or differentiation periods. Using this model, it is possible to create different local culture niches to drive osteoblasts to different cellular developmental stages, which alter the gene expression and dictate the downstream extracellular matrix biosynthesis, maturation and mineralization. Although aggregation is accepted to accelerate mineralization, this manuscript offers an insight into how aggregate size and culture duration affects mineralization and the variation of mineral composition. The model described in this study has also allowed for the qualitative visualization and quantitative comparison of mineral development and spatial distribution within different sized aggregates over time by FTIR and other techniques (manuscript in preparation) and may allow us to investigate the regulatory factors at different developmental phases separately and independently. Eventually, the large aggregate cell cultures may potentially be used for the rapid generation of bone graft material for reliable bone fusion in orthopedic surgery.

## Electronic supplementary material

Additional file 1: **The movie recording the outgrowth processing of aggregates for 20 hour after the aggregates being cultured for 48 hours on suspension plates.** Some cells migrated out or proliferated from the periphery of large aggregates and formed a sunflower-shaped morphology, whilst small aggregates turned into planar cell plaque and lost the dense central part during the outgrowth stage. (ZIP 2 MB)
